# The progeria research foundation 10^th^ international scientific workshop; researching possibilities, ExTENding lives – webinar version scientific summary

**DOI:** 10.18632/aging.202835

**Published:** 2021-03-17

**Authors:** Leslie B. Gordon, Kelsey Tuminelli, Vicente Andrés, Judith Campisi, Mark W. Kieran, Lynn Doucette, Audrey S. Gordon

**Affiliations:** 1 Department of Pediatrics, Division of Genetics, Hasbro Children’s Hospital and Warren Alpert Medical School of Brown University, Providence, RI 02903, USA; 2 Department of Anesthesiology, Perioperative and Pain Medicine, Boston Children’s Hospital and Harvard Medical School, Boston, MA 02115, USA; 3 The Progeria Research Foundation, Peabody, MA 01961, USA; 4 Centro Nacional de Investigaciones Cardiovasculares (CNIC), Madrid 28029, Spain; 5 Centro de Investigación Biomédica en Red de Enfermedades Cardiovasculares (CIBERCV), Madrid 28029, Spain; 6 Buck Institute for Research on Aging, Novato, CA 94945, USA; 7 Biosciences Division, Lawrence Berkeley National Laboratory, Berkeley, CA 94720, USA; 8 Invited Expert, Yardley, PA 19067, USA

**Keywords:** progeria, laminopathy, lamin A, aging, atherosclerosis

## Abstract

Progeria is an ultra-rare (prevalence 1 in 20 million), fatal, pediatric autosomal dominant premature aging disease caused by a mutation in the *LMNA* gene. This mutation results in accumulation of a high level of an aberrant form of the nuclear membrane protein, Lamin A. This aberrant protein, termed progerin, accumulates in many tissues and is responsible for the diverse array of disease phenotypes. Children die predominantly from premature atherosclerotic cardiovascular disease. The Progeria Research Foundation’s 10^th^ International Scientific Workshop took place via webinar on November 2 and 3, 2020. Participants from 30 countries joined in this new, virtual meeting format. Patient family presentations led the program, followed by updates on Progeria’s first-ever application for FDA drug approval as well as initial results from the only current Progeria clinical trial. This was followed by presentations of unpublished preclinical data on drugs in development targeting the disease-causing DNA mutation, the aberrant mRNA, progerin protein, and its downstream effector proteins. Tying bench to bedside, clinicians presented new discoveries on the natural history of disease to inform future clinical trial development and new Progeria aortic valve replacement procedures. The program engaged the Progeria research community as a single unit with a common goal – to treat and cure children with Progeria worldwide.

## INTRODUCTION

The Progeria Research Foundation 10^th^ International Scientific Workshop, “Researching Possibilities, ExTENding Lives”, was originally slated to be an in-person gathering, as it has been every two years since 2001. These meetings are intense, 2.5-day events that essentially sequester a group of highly motivated basic and clinical researchers who are intent on understanding Hutchinson-Gilford Progeria Syndrome (HGPS or Progeria) and discovering treatments and the cure for affected children (HGPS or Progeria). HGPS is an ultra-rare (prevalence 1 in 20 million), autosomal dominant premature aging disease. In 90% of cases, Progeria is caused by a single C>T mutation at position 1824 in the *LMNA* gene, causing increased use of a cryptic splice site within exon 11 [1, 2]. The resulting transcript lacks 150 bases, translating into a preprotein missing 50 amino acids, which causes aberrant post-translational processing. The end result is a dominant negative disease-causing inner nuclear membrane protein called progerin, which, unlike normal lamin A, remains permanently farnesylated [2]. Without progerin-targeted treatment, children with Progeria die from heart attacks or strokes at an average age of 14.5 years following premature, accelerated atherosclerosis [3, 4]. The mission of The Progeria Research Foundation (PRF) is to find the cause, treatments and cure for Progeria and its aging-related disorders, including heart disease (www.progeriaresearch.org). All scientific projects presented at the workshop were supported in part by PRF’s grant program.

With record-high numbers of both peer-reviewed publications on Progeria and requests for cell lines and samples from the PRF Cell and Tissue Bank for HGPS preclinical explorations, this field is on the verge of major advancements. Indeed, with the first-ever FDA drug approval for HGPS achieved just 2 weeks after this workshop [5], Progeria joins only 10% of the more than 7,000 rare diseases with an FDA-approved drug [6]. Treatment with the farnesyltransferase inhibitor lonafarnib (now marketed as Zokinvy™) demonstrated an average 2.5-year survival benefit over an untreated control group derived from the PRF International Patient Registry [4]. It also improved cardiovascular status and had a relatively well tolerated side effect profile [7, 8]. With 2.5 years representing an average 17% lifespan increase, Zokinvy is a beacon of hope that tells us the disease can be pushed towards health – and that we can and must discover even better treatments and the cure for Progeria.

With COVID 19 bringing the planned in-person 2020 PRF scientific meeting to a screeching halt, the organizers used a webinar format. Knowing it would be impossible to re-create an in-person meeting, the goals were to keep our scientists engaged, inspired, and up-to-date with the newest unpublished data. The feeling of community was of paramount importance and could not wait another year or two for an in-person experience.

**Figure 1:**
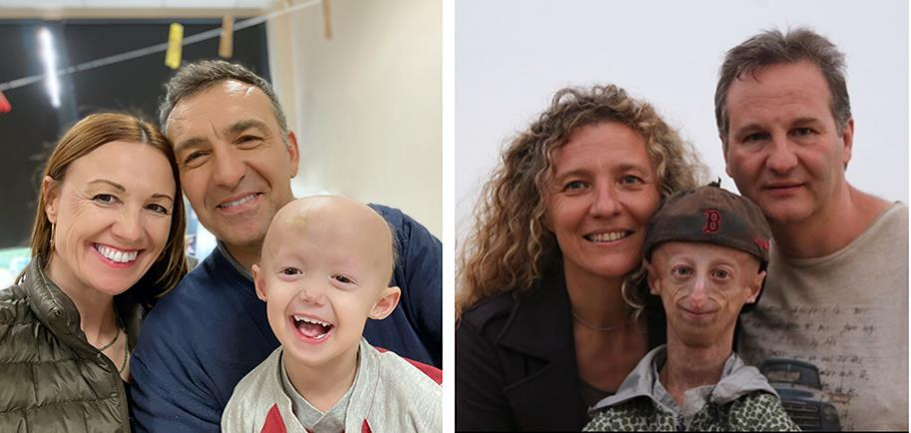
**Two patient families presented their views on life with Progeria in the opening session of the 2020 PRF International Scientific Workshop.** (Informed consent has been obtained).

**Figure 2:**
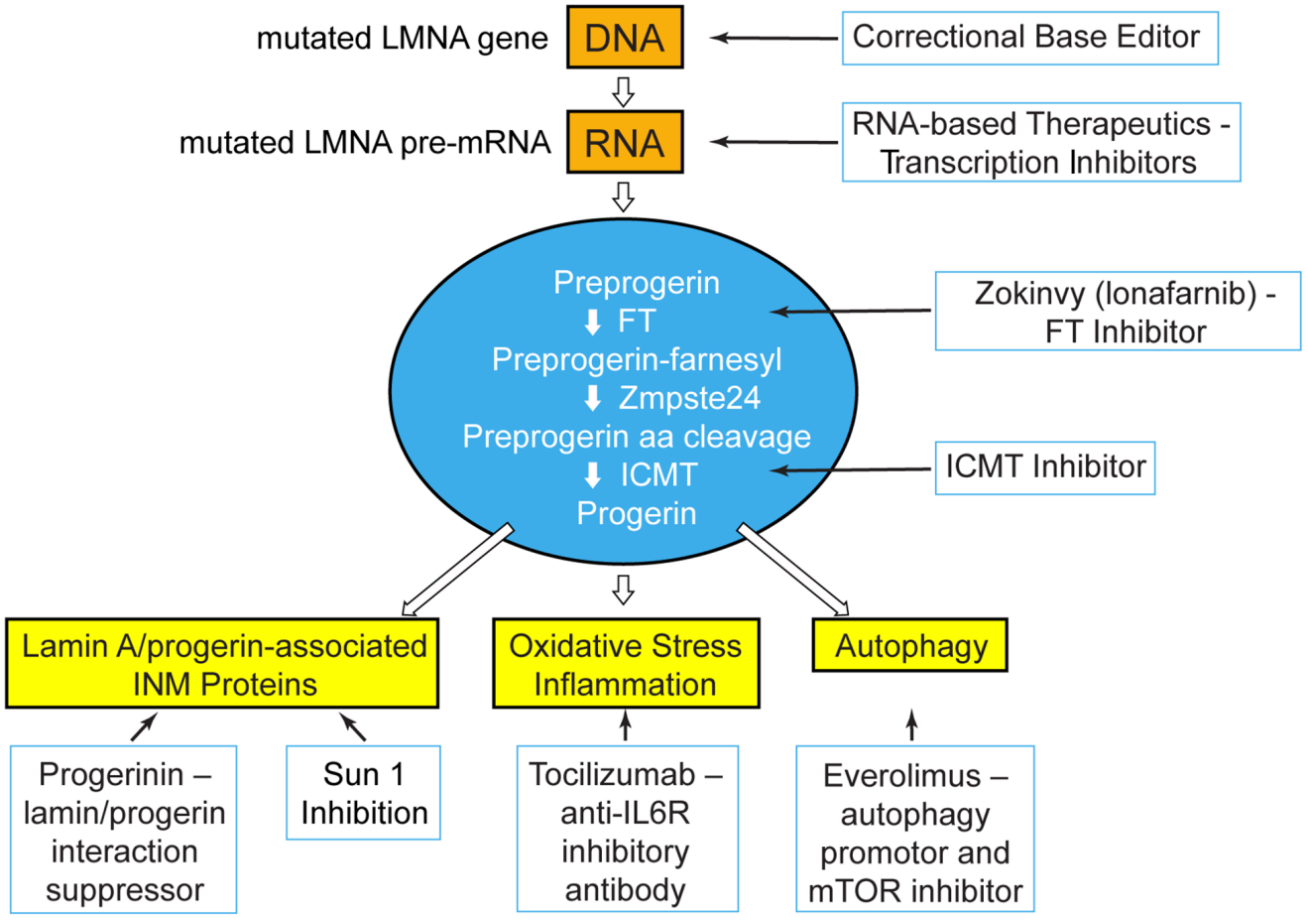
**At the PRF International Scientific Workshop, unpublished data were presented for treatment strategies that blanket the pathways upstream, at, and downstream of the disease-causing protein, progerin.** Treatment targets: genetic and transcriptional shown in orange; post-translational processing shown in blue; disease effectors downstream of progerin shown in yellow. FT=farnesyltransferase; ICMT=Isoprenylcysteine Carboxyl Methyltransferase; INM=inner nuclear membrane; IL6R=interleukin 6 receptor; mTOR=mechanistic target of rapamycin.

The meeting was limited to two 3-hour sessions over 2 days. Because Progeria is ultra-rare, outside of the PRF workshops most scientists have no opportunity to meet the very children they are working to save. Thus, it was most fitting to first hear from children and young adults with Progeria, and their families (Figure 1). The subsequent scientific sessions were designed to work backwards, first presenting data on Zokinvy, the trial medication that was submitted for FDA approval, as well as the ongoing Progeria clinical trial of Zokinvy in combination with the mTOR inhibitor, everolimus (Figure 2). This was followed by presentations on promising treatments targeting various preclinical phases in each of 3 areas of drug development (Figure 2): 1) influencing progerin protein, or proteins that interact with or act downstream of progerin; 2) inhibiting progerin mRNA production with RNA therapeutics; and 3) correcting the *LMNA* mutation by genome editing. The final session maximized communication between basic scientists and clinical investigators by exploring the newest research on the natural history of cardio-neurovascular disease in HGPS, and how we can use this clinical knowledge to improve trial outcomes and assessments.

## Creating crucial connections between patients and researchers

The lifespan and quality of life of the Progeria population are changing, at least in part due to advances in Progeria research [7, 8]. Eloquent speakers living with Progeria inspired the very researchers who are their greatest hope for the future. Two families presented different, important perspectives. The first to speak was the family of Sammy from Italy. Sammy has been living with Progeria since before PRF existed, before the gene was identified, and before any clinical trials were conducted. More children with Progeria are now entering young adulthood, something that was rare prior to the past decade [4]. Sammy has graduated college and is now pursuing a post-graduate degree in molecular biology. His presentation was captivating, as he described his current status as an advanced degree student, his daily life with friends and family, and his aspirations for the future. The second family came to Progeria with the more recent perspective that many families have. Their child, Alexandra (Spain), is 4 years of age. In their experience, diagnosis and clinical trial participation were available to them as soon as Progeria was recognized as a possibility (which is occurring at a much younger age today than for children in the past). To them, the prospects for their child receiving a life-altering therapy or cure in her lifetime seem highly achievable, but only with an intensely focused, high-priority scientific and medical effort.

## Updates on current and previous clinical trial therapies

Zokinvy is a farnesyltransferase inhibitor that blocks addition of a farnesyl lipid moiety onto pre-progerin, thus preventing progerin from associating with the inner nuclear membrane where it effects much of its damage. Several weeks after this workshop, Zokinvy was approved for use in Progeria, its first and only approved indication. David Cory (Eiger BioPharmaceuticals, Inc., Palo Alto, CA) discussed the history of lonafarnib development from basic science to FDA drug application [5]. Lonafarnib was initially discovered and developed by Schering-Plough Research Institute and then Merck Research Labs as a potential cancer therapeutic, however it failed to show robust clinical activity in late-stage cancer trials. Schering-Plough and Merck had been providing drug to PRF for initial preclinical and subsequent clinical studies. Merck licensed lonafarnib to Eiger in 2018 who continued and expanded the collaboration with PRF. Eiger is also investigating lonafarnib for use in a non-HGPS indication, Hepatitis Delta.

The only ongoing clinical trial for HGPS combines lonafarnib with everolimus [9] (Monica Kleinman, MD, Boston Children’s Hospital, Boston, MA). Everolimus is an analogue of the mTOR inhibitor rapamycin and is FDA-approved for the treatment of various cancers as well as for children and adults with the genetic condition tuberous sclerosis. Everolimus and/or rapamycin have demonstrated benefit in HGPS cells in culture and lamin-deficient mouse models [10–14], and preclinical data suggest that everolimus increases clearance of progerin through an elevated proteolytic degradation process termed autophagy. Based on these findings, everolimus is being repurposed to explore its possible clinical benefit in Progeria. With the phase 1/2 trial having completed accrual, the recommended phase 2 dose was identified based on its safety and tolerability profile in this patient population and overlaps with the dose being used in other non-Progeria indications. Enrollment in the phase 2 component of the trial is also now complete, with 60 children from 27 countries enrolled in phase 2 for efficacy assessments. Efficacy will be assessed as change in carotid-femoral pulse wave velocity, a vascular health measure, as well as rate of weight gain. The single site trial requires in-person visits to Boston, some of which have been delayed due to COVID 19. With the promise of COVID 19 vaccination on the horizon, the trial is expected to finish in 2021.

## Post-translational treatment strategies

Of the strategies aimed at treating HGPS at the post-translational level, a small molecule called progerinin appears to be the most advanced, with a first-in-human adult healthy volunteer clinical trial currently underway (Minju Kim, PhD and Bum-Joon Park, PhD, Pusan National University, Korea) [15]. Progerinin is an optimized derivative of JH4, a molecule that inhibits the interaction between lamin A and progerin [16]. Progerinin potently reduced expression of progerin and ameliorated nuclear anomalies in cultured HGPS fibroblasts. In a HGPS mouse model, progerinin increased body weight and extended lifespan by 10 weeks over excipient-treated controls when dosed at 20 mg/kg twice weekly, compared with lifespan extension of only 2 weeks in a lonafarnib-treated control arm.

Repurposing drugs approved for non-HGPS indications can overcome tremendous hurdles involved with early drug development. Giovanna Lattanzi, PhD (CNR Institute of Molecular Genetics, Bologna, Italy) presented studies on neutralizing the pro-inflammatory cytokine interleukin 6 (IL6) by repurposing tocilizumab, a drug approved for treating human arthritis [17]. Oxidative stress likely plays a downstream role in tissue damage in HGPS, and IL6 plays a major role in promoting the oxidative stress response. Tocilizumab is a humanized monoclonal antibody against the interleukin-6 receptor (IL-6R). Neutralization of IL6 with tocilizumab treatment reduced both progerin levels and nuclear defects in HGPS cells. Moreover, progeroid mice treated with tocilizumab showed skin improvements, weight gain, better locomotor activity, and lifespan extension.

Two proof-of principle approaches were presented in early stages of development. The first focused on inhibiting S-isoprenylcysteine O-methyltransferase (ICMT), the enzyme necessary for the last step in the post-translational processing of progerin (Martin Bergö, PhD, Karolinska Institutet, Stockholm, Sweden) [18]. Cultured cells displayed improvements including reduced cellular senescence markers, increased proliferation, and reduced reactive oxygen species when treated with an ICMT inhibitor. A second strategy is inhibition of the interaction between lamin A and the inner nuclear membrane protein SUN1 (Colin Stewart, PhD, Institute of Medical Biology, Immunos, Singapore) [19]. SUN1 also anchors a protein called nesprin1 to the nuclear envelope, and reduced nesprin1 protein levels were observed in SUN1-deficient HGPS cell lines. HGPS mice were genetically modified to express either reduced amounts of ICMT or SUN1 protein. In both cases, mice displayed significantly extended lifespans. Both laboratories are now focused on discovering and developing drugs that target these pathways.

## RNA therapeutics

Because the mutations causing HGPS are well-defined, HGPS is well-positioned for the development of RNA and DNA-based therapies. Proof of principle for both strategies has been accomplished in both cellular and mouse models of HGPS [20, 21]. For RNA-based antisense therapy, efficacy depends on delivery of short single-stranded RNAs with sequence complementarity to the mRNA encoding progerin. Efficient inhibition of progerin mRNA translation is dependent on both the length and sequence of the antisense RNA. For *in vivo* use, it is also critical that the antisense mRNA has chemical modifications to ensure both stability in plasma and efficient delivery to key affected tissues. Two groups have systematically explored the relevant regions of the mutated HGPS *LMNA* gene using different antisense therapeutics in search of optimal inhibition of the expression of the pathogenically spliced progerin transcript. In the first talk, Francis Collins, MD, PhD (National Institutes of Health, Bethesda, MD) showed that treating progeroid mice with a drug named SRP2001 reduced progerin mRNA and protein expression in the aorta, the main tissue of interest, as well as in other tissues. This was associated with less aortic wall pathology and over 60% improved survival [22]. In the second talk, Tom Misteli, PhD (National Cancer Institute, NIH, Bethesda, MD) showed 90 – 95% reduction of progerin mRNA in different tissues; however, progerin protein reduction was most effective in liver, with less effect in the heart and aorta. Combination treatment with RNA therapeutics and Zokinvy reduced progerin protein levels in liver and heart more strongly than either single treatment [23].

## DNA base editing

By far the most dramatic improvements in disease phenotypes observed in an HGPS mouse model came from cutting-edge experiments showing gene correction via base editing (David R. Liu, PhD, Howard Hughes Medical Institute, Broad Institute, Cambridge, MA), published several months after the workshop [24]. The mutation causing classic HGPS is a single C>T mutation at position 1824 in the *LMNA* gene. The research group created targeted adenine base editors that use an adenosine deaminase to convert A/T base pairs to G/C base pairs without requiring double-strand DNA breaks. The group then employed this method to reverse the mutation in HGPS cells, transmuting them to wild type. Using adeno-associated virus 9 (AAV9) for drug delivery, they achieved 90% correction to wild-type lamin A 10-20 days following infection of patient fibroblasts. This resulted in reduced levels of both mis-spliced RNA transcript and toxic progerin protein, and rescue of nuclear blebbing. Treatment of HGPS mice with a single retro-orbital injection of AAV9 at day 3 of life yielded a 1.8-fold increase in average lifespan, whereas intravenous injection at day 14 of life resulted in an average 2.5-fold lifespan extension. Vessel wall abnormalities, spine curvature, activity levels and fur loss were also greatly improved. Importantly, the group found no off-target editing, though several mice developed hepatocellular carcinoma, a known side effect in mice receiving AAV therapies.

## Clinical research for trial evaluations and clinical care

An important question for all proposed therapies centers around the extent of benefit that can be achieved when treatment starts early versus later in disease progression. Children with HGPS enrolled in clinical trials with lonafarnib exhibited vascular wall improvement at all ages and disease stages [7, 25]. However, it is not known whether this will be the case for other therapies, or if the improvements will correlate with the extent of progerin reduction in different tissues. To address this issue, a new mouse model with reversible progerin expression was unveiled (Vicente Andrés, PhD, CNIC, CIBEREV, Madrid Spain). The model recapitulates the main features of HGPS and can be used to ask what happens when progerin is dampened or eliminated at different ages and/or in different tissues. Can damage be reversed? How late can we start treatment and still have a therapeutic benefit? Shall we target all tissues, or would tissue-specific therapies be effective? The model may recapitulate real-life scenarios since children with HGPS have historically initiated treatment at widely different ages and stages of disease.

The role of clinical research into the natural history of disease in Progeria cannot be overstated. It provides essential fuel for basic scientists to understand how to best measure drug effects in cellular and animal models, and defines which disease measures reflect clinical disease status for clinical trials; all facilitating bench to bedside success. Eighty percent of people with HGPS die of heart failure or heart attacks [4]. To that end, the Progeria cardio-neurovascular team at Boston Children’s Hospital in Boston, MA (Leslie Smoot, MD and Ashwin Prakash, MD) and Brigham and Women’s Hospital (Marie Gerhard-Herman, MD and Srinivas Mukundan, MD, PhD) incorporate long-term natural history investigations as part of ongoing Progeria clinical trials [7, 25, 26]. Overall their goals are to discover objectively evaluable vascular and cardiac abnormalities, assess which contribute to mortality, and identify quantitative and reproducible measurements that could be used as readouts in clinical trials to monitor treatment efficacy. Adapting new technological advances as well as new discoveries on meaningful clinical correlates in the aging population to the field of Progeria means that this is a highly dynamic endeavor, with opportunities for new discovery constantly arising. Here, echocardiography was used to evaluate cardiac structural and functional abnormalities across trial participants aged 1-25 years, and longitudinally over multiple trial visits. Though some aspects of cardiac disease such as aortic and mitral valve stenosis and calcification are important to clinical decline, they occurred primarily in the second decade of life, which presents challenges to using them as short or medium-term trial outcome measures. However, left ventricular diastolic dysfunction (LVDD) arose as the earliest and most common abnormality, beginning at age 2-3 years, with the extent of dysfunction increasing in each patient as he or she aged [26]. This echocardiographic parameter measures an early sign of disease of the left ventricle, reflecting an inability of the heart chamber to properly contract and relax during the cardiac cycle. A major factor causing this dysfunction could be myocardial fibrosis, seen in HGPS mouse and pig models, and human autopsies [27–32]. Since left-sided heart failure is a major cause of morbidity and mortality in HGPS, the identification of this objective measure of disease should be prioritized to evaluate possible treatment benefit. Analysis of whether this deficit can be reversed versus slowed or halted will be crucial to inform trial design. In addition to LVDD, newer technology in echocardiographic imaging has facilitated a highly sensitive study of myocardial deformation in different sections of the left ventricular muscle [33]. Future studies will evaluate LVDD in combination with myocardial deformation in hopes of better identifying clinically significant patient outcome measures.

Vasculature abnormality contributes to LVDD, whereas vessel stiffening and atherosclerotic plaques create stress on the heart. However, examining the effects of small, fibrotic, often plaque-laden carotid arteries on the brain is also important for understanding stroke risk, especially in children and young adults now living longer due to Zokinvy therapy. Studying the medium and small vessels using both carotid ultrasound and magnetic resonance imaging of the brain has thus far provided measures of vascular disease that have been translated into trial outcome measures [2, 7, 25, 34, 35]. These include structural evaluation using echobrightness of the carotid vessel wall, a reflection of medial and adventitial fibrosis, as well as carotid-femoral pulse wave velocity, a measure of vessel stiffness. In both of these measures, children with Progeria display clinically consequential abnormalities that can be partly alleviated by Zokinvy therapy. In both aging adults and in Progeria, collateral vessels develop in response to a relative lack of blood supply to the brain through the major vasculature [36]. These vessels provide essential blood supply but are generally fragile and more susceptible to bleeding. Though few deaths in HGPS are attributed to strokes, stroke risk is estimated at up to 1.75/year in children with HGPS, and can result in significant morbidity [34]. Development of collateral vessels precedes strokes in most children with HGPS, and can be identified at a young age [34, 35]. Both the extent and pattern of collateral vasculature in the brain are being studied as possible predictors of clinical outcome, and early studies suggest possible improvement in the form of improved flow in native vessels along with regressing collateral vessels with Zokinvy therapy.

The end stages of HGPS are a dramatic premature recapitulation of advanced atherosclerosis in the elderly, but with some important additional challenges. Whereas elderly patients can often receive cardiac intervention, those with Progeria are much smaller, often precluding cardiac intervention. In 2019, a new, smaller-sized aortic valve was approved in Europe and the US, opening the possibility of performing high risk aortic valve replacement in a subset of the relatively larger patients with HGPS. Two cardiac surgeons have successfully performed Transcatheter Aortic Valve Implantation (TAVI) in patients with severe aortic stenosis resulting in progressing heart failure - one in a young man with HGPS (Dr. Francesco Musumeci, MD, S. Camillo Hospital Roma, Azienda Ospedaliera San Camillo Forlanini, Italy) [37], and the other in a woman with the Progeroid Laminopathy called Mandibuloacral dysplasia (MAD; Pinak Shah, MD, Brigham and Women’s Hospital, Boston, MA). Though the patient with MAD underwent the procedure through the more traditional femoral artery route, the procedure was performed transapically for the 23-year-old patient with HGPS because access through the femoral artery was blocked by arterial plaques. The procedure was successful for each patient, and follow-up at 1 and 2 years demonstrated improved cardiac function and quality of life. These successful procedures were the first of their kind and, although not all children with HGPS will be of adequate size to receive a TAVI, this opens the door to the possibility for a subpopulation of those with HGPS to receive significant symptomatic relief and possibly longer lifespans.

## Summary

The biannual PRF scientific workshop is an important milestone for the small but highly influential Progeria scientific community. Though the COVID 19 pandemic has affected the ability of scientists to conduct their work, travel, and gather in person, the online gathering showed the perseverance of Progeria scientists who continue to make progress despite significant obstacles. Viable treatment targets both upstream and downstream of progerin are actively being pursued by the research community. Research on traditional small molecule drug and biologic therapy development and repurposing, RNA therapeutics, as well as genomic editing have demonstrated benefit in HGPS cells and mouse models. Moreover, innovative animal models are being generated that will allow Progeria scientists to address new relevant questions in translational research. The field continues to grow in scope and sophistication as the search for effective treatments and the cure continues. Translating these into clinical trials with favorable risk/benefit ratios, designing trials that take into consideration the new standard of care therapy, Zokinvy, and accurately assessing benefit in patients are the challenges that will garner success in further improving quality of life and longevity for children with Progeria in the next wave of therapeutic endeavors.
